# Kinoscope: An Open-Source Computer Program for Behavioral Pharmacologists

**DOI:** 10.3389/fnbeh.2017.00088

**Published:** 2017-05-12

**Authors:** Nikolaos Kokras, Dimitrios Baltas, Foivos Theocharis, Christina Dalla

**Affiliations:** ^1^Department of Pharmacology, Medical School, National and Kapodistrian University of AthensAthens, Greece; ^2^First Department of Psychiatry, Medical School, National and Kapodistrian University of AthensAthens, Greece

**Keywords:** computer program, behavioral pharmacology, forced swim test, elevated plus maze, novel object recognition, scoring behavior

## Abstract

Behavioral analysis in preclinical neuropsychopharmacology relies on the accurate measurement of animal behavior. Several excellent solutions for computer-assisted behavioral analysis are available for specialized behavioral laboratories wishing to invest significant resources. Herein, we present an open source straightforward software solution aiming at the rapid and easy introduction to an experimental workflow, and at the improvement of training staff members in a better and more reproducible manual scoring of behavioral experiments with the use of visual aids-maps. Currently the program readily supports the Forced Swim Test, Novel Object Recognition test and the Elevated Plus maze test, but with minor modifications can be used for scoring virtually any behavioral test. Additional modules, with predefined templates and scoring parameters, are continuously added. Importantly, the prominent use of visual maps has been shown to improve, in a student-engaging manner, the training and auditing of scoring in behavioral rodent experiments.

## Introduction

Behavioral analysis in preclinical neuropsychopharmacology relies on the accurate measurement of animal behavior (Kokras and Dalla, 2014; Kokras et al., 2015). Appropriate operating procedures and intensive experimenter training may influence or determine behavioral performance (Chesler et al., 2002; Sousa et al., 2006). Advances in computer science allowed the development of elaborate software which records animal behavior, often with a high degree of automation, taking advantage of intelligent algorithms and image tracking technologies (Noldus, 1991; Noldus et al., 2000, 2001; Zimmerman et al., 2009). However, those commercially available solutions have a high purchasing cost. In addition, automated algorithms may provide better scoring than humans in some cases (Desland et al., 2014) but may also provide less accurate and detailed analysis than humans in certain other cases, as in the forced swim test (distinguishing fine transitions between swimming, climbing and immobility behaviors) and novel object recognition (distinguishing active interest toward the object vs. near vicinity of the animal's head). Several attempts have been done over the last 20 years to develop open-source or freely available computer programs for scoring animal behavior (Moraes and Ferrarezi, 1997; Ottoni, 2000; Taiwanica, 2000; Patel et al., 2006; Poirrier et al., 2006; Aguiar et al., 2007; Blumstein and Daniel, 2007; Otero et al., 2010; Crispim Junior et al., 2012; de Chaumont et al., 2012; Telonis and Margarity, 2015; Friard et al., 2016). Some of those attempts resulted in outdated and probably not under active development computer programs, some were focused on specific models that could not be easily modified for other settings, and some resulted in elaborat solutions that required a significant investment in human resources to develop, adapt and operate. Large-scale behavioral laboratories routinely invest in high-cost commercially available solutions and are also willing to invest human resources in developing in-house esoteric approaches. However, it is not rarely the case that a research team needs a straightforward computer aid to perform a widely-used behavioral test for a specific project. Additionally, in those cases where human scoring is required or desired, there is difficulty in training students and staff members to accurately and reproducibly score animal behavior. This is of paramount importance, as inaccurate scoring by improperly trained personnel may contribute to non-reproducible results. Few computer programs place as priority an interface that facilitates correct training of students and staff. In this context, we have developed a versatile and expandable software package with the aim to provide a ready and easy to use platform for behavioral analysis scoring and a platform through which training in behavioral pharmacology scoring can be facilitated and controlled.

## Description of the system

**Figure 1:**
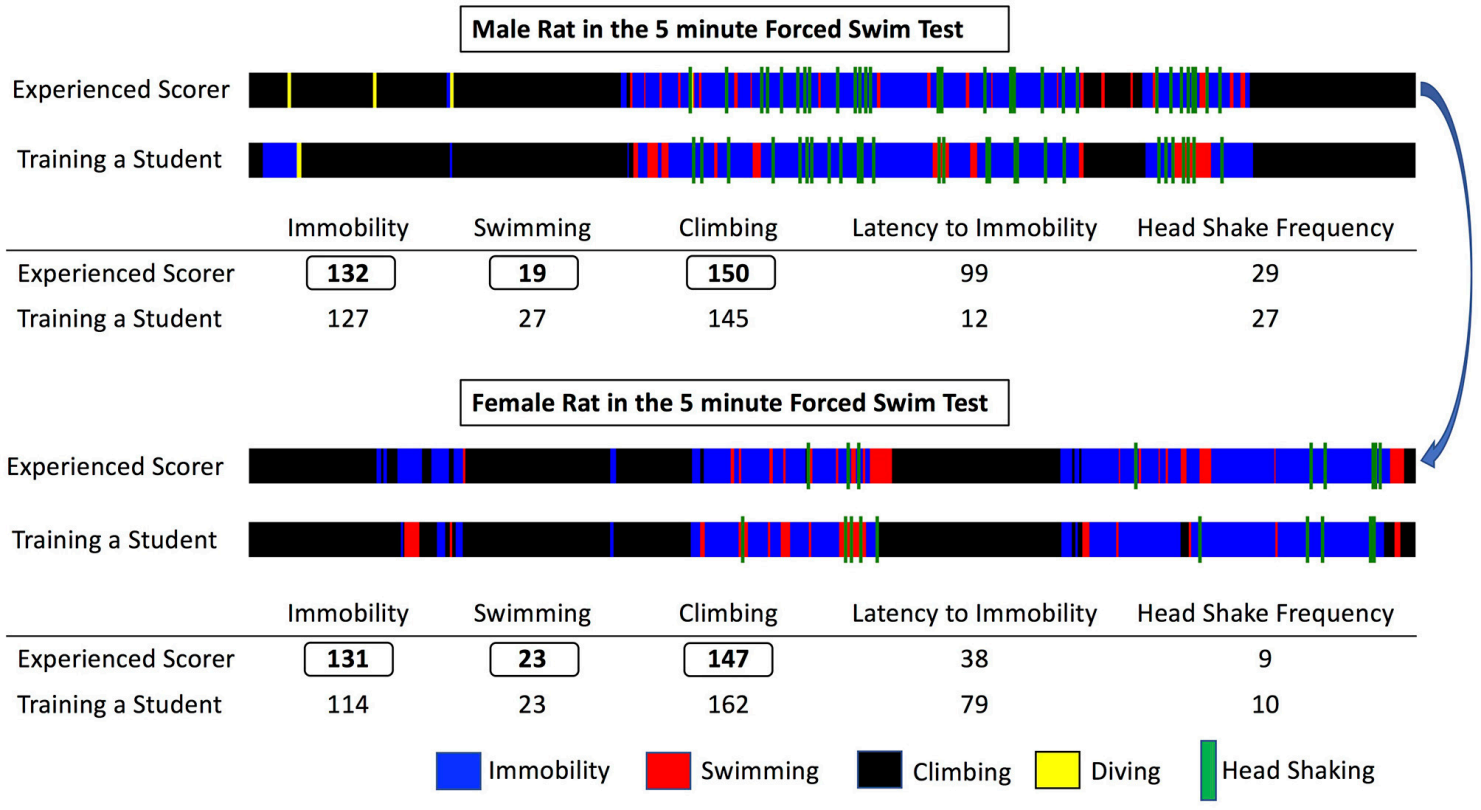
**Representative visual maps produced after scoring a male (top)** and a female **(bottom)** rat during Forced Swim Test. The total length of the visual bar corresponds to the test duration (5 min) and each behavior scored is depicted with a designated color, its time of appearance and its duration. Note that both animals have almost identical total duration of immobility, swimming, and climbing, however the organization in time of the observed behaviors differs significantly between the male and the female rat. Also, note the slight differences between the experienced scorer and a trainee, the latter performing the scoring in a satisfactory way, if examining only the total scores, but still committing some errors when inspecting the visual maps. By comparing the produced visual maps and discussing the animal's performance training can be facilitated in an engaging way and reproducibility can be enhanced.

**Figure 2:**
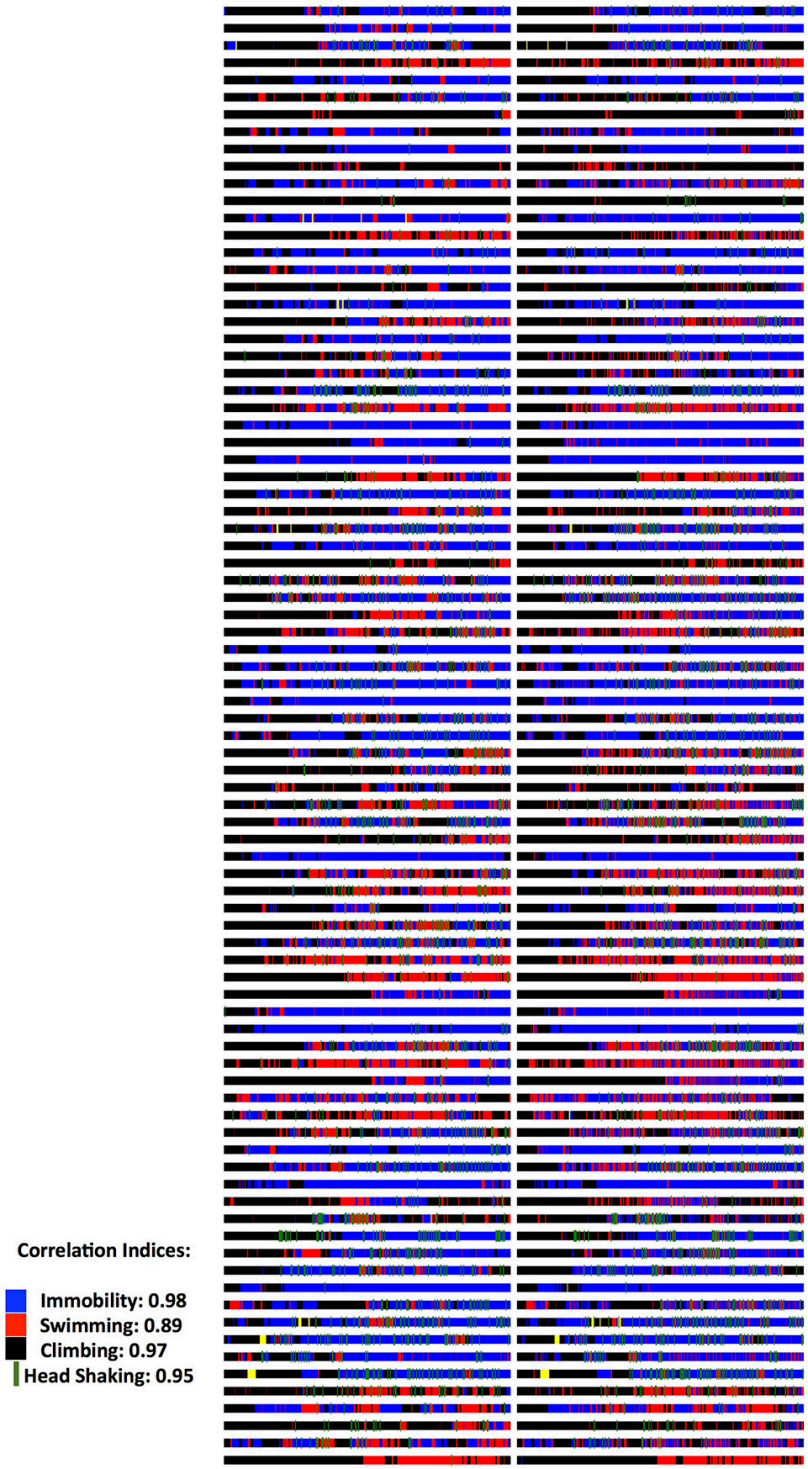
**Validation data on Forced Swim Test (FST) Scoring**. Two experienced raters, after having trained with the Kinoscope program, scored blindly, and independently male and female rats in the 5 min second session of the two-sessions FST. Each animal is represented in a separate row and on each column, the scoring pattern from each blind and independent rater can be seen. Correlation indices were 0.85 for number of recorded behavioral events, 0.98 for immobility behavior (blue color) and 0.90 for immobility latency, 0.89 from swimming (red color), 0.97 for climbing behavior (black color), 0.95 for head shaking frequency (green color). All correlations were highly significant (*p* < 0.001) as indicated by Pearson's two-tailed test. Full data published in Kokras et al (Kokras et al., 2015). Raw images from Kinoscope were put in order and collated together using ImageJ/Fiji (Schindelin et al., 2012; Schneider et al., 2012).

**Figure 3:**
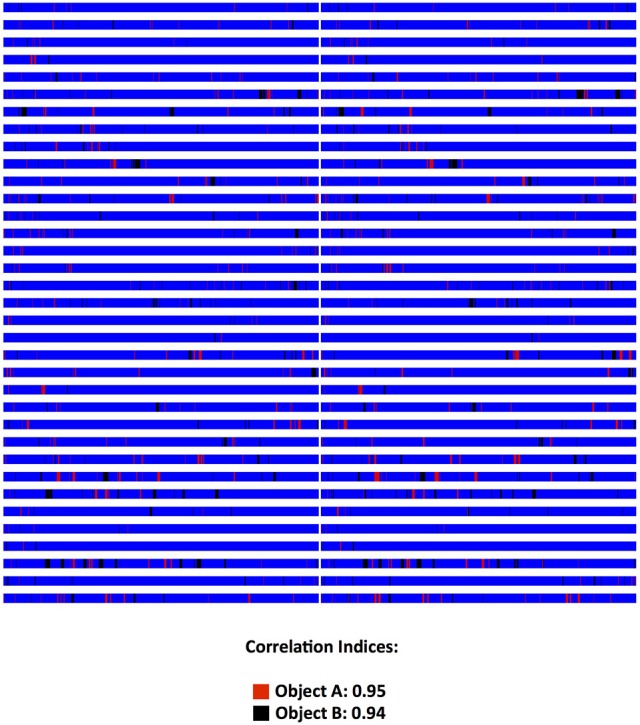
**Validation data on Novel Object Recognition Scoring (NOR) from a yet unpublished experiment**. Two novice student raters, after having trained with the Kinoscope program, scored blindly, and independently male and female rats in the 5 min second trial of a two-trial NOR. Each animal is represented in a separate row and on each column, the scoring pattern from each blind and independent rater can be seen. Correlation indices were 0.90 for number of recorded behavioral events, 0.95 and 0.91 for Object A (red) time and frequency respectively, and 0.94 and 0.87 for Object B (black) time and frequency respectively. Time in general area of the open field is depicted in blue color. All correlations were highly significant (*p* < 0.001) as indicated by Pearson's two-tailed test. Raw images from Kinoscope were put in order and collated together using ImageJ/Fiji (Schindelin et al., 2012; Schneider et al., 2012).

**Figure 4:**
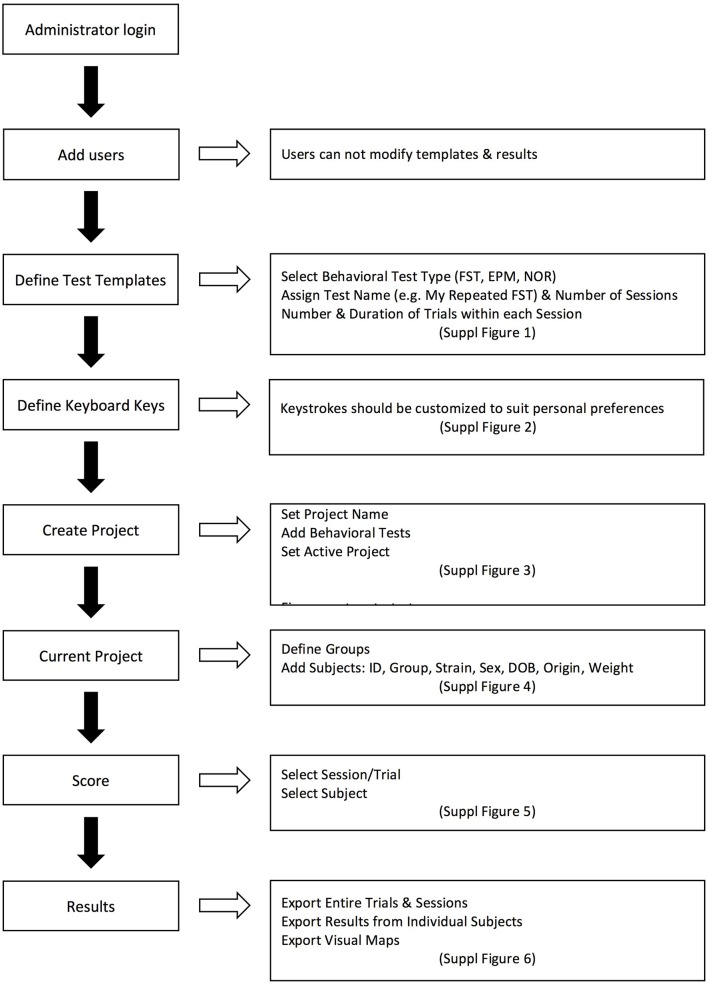
**Representative workflow of using the Kinoscope, with references to Supplemental Figures (S1–S6) (screenshot figures) explaining each step of the procedure**.

The program is developed in Visual C# and is released under the GNU General Public License, version 3 (GPL v3) (GNU, 2007). It is compatible with personal computers able to run MS Windows XP© or later operating system versions (through MS Windows 10©). There are no other minimum system requirements; hence the program can run on a variety of computers, even outdated. To run the program however, the computer must have installed the MS.NET Framework version 4.0© library extension (Platt, 2002), which is freely available from the manufacturer and which is widely used in many software packages for MS Windows© operating systems. Data generated by the program are stored in a relational database compatible with different engines (SQLite, MySql, MS-SQL, etc). The program, for the time being, contains pre-installed templates for three popular behavioral tests, namely the Forced Swim Test (Slattery and Cryan, 2012), Novel Object Recognition (Akkerman et al., 2012) and the Elevated Plus Maze (Walf and Frye, 2007). Those can be adapted to suit the specific needs of each researcher with regards to number and duration of sessions/trials. This is particularly useful if for example FST is performed as a single or dual session, or when NOR is performed in multiple trials on each day and on multiple sessions along several days. In addition, the concept of having experiments organized and correctly archived is best served by organizing “projects” (protocols): each one can be named accordingly and be operated independently, contains any number of custom-tailored behavioral tests and contains a database of subjects (experimental animals) that will be subjected to one or more behavioral tests according to the “project” (protocol). Also, according to the details that researchers wish to include in the database and in the exported files, descriptors of experimental groups (vehicle, treatment, stress etc.) and of subjects (i.e., sex, age, origin etc.) can be determined. By providing a definition of experimental groups and subjects, a more organized database can be obtained and a cleaner output can be exported, facilitating auditing, archiving and later retrieval. The assignment to groups can also be done at a later stage, even after the scoring process, for those protocols that require subjects to be assigned to groups not randomly but based on behavioral or other criteria. Scoring procedure relies on the researcher indicating with the appropriate keystrokes the observed behavior either when observing the live animal or a video recording of its behavior. The program comes with a predefined set of key mappings, which can be modified, as it is usually done in similar software solutions (Blumstein and Daniel, 2007; Friard et al., 2016). A key strength of the software is the assignment of color codes to each behavioral element, thus providing a visual aid for the researcher while scoring. During scoring a progress bar indicates the elapsed and remaining time along with the observed behaviors in their designated color codes. This allows for trainees to understand how an experienced observer scores and assists them in learning. Furthermore, visualizations representing the organization (time sequence) of observed behaviors can be exported as images. Those are exported in separate files for each animal (in png format) and separately from the main results output (which is exported in a spreadsheet compatible document). This functionality of producing “visual maps” is partially implemented in other programs as well (de Chaumont et al., 2012) and has been proven particularly useful in two ways: firstly, differences in the organization of behaviors between different animals can be easily highlighted and secondly, a trainer and a trainee can visually compare their scoring and discuss possible discrepancies (Figure 1). From our experience (Kokras et al., 2014, 2015, 2017), on rater-independent and blind scoring, the intra- and inter-rater agreement of observers previously trained with Kinoscope reaches a correlation of well beyond *r* = 0.9, thus significantly increasing the validity and reproducibility of animal behavior data (Figures 2, 3). Such correlation indices are higher than those previously observed in our research team, when scoring was performed without Kinoscope. Upon completion of the manual scoring and according to the type of behavioral test, certain predefined measures are automatically calculated beyond the primary measurements (e.g., Latencies for FST, % in Open Arms in EPM, Discrimination & Preference Indices in NOR). Results are finally exported, either for the whole trial/session or for selected time segments, in csv (comma-separated values) format, which can then be imported in most spreadsheet and statistical software packages. A simplified workflow of the entire use of the system is summarized in Figure 4 with references to a series of Supplemental Figures (S1–S6)/Screenshots.

## Conclusion

Accurate behavioral analysis remains of paramount importance in preclinical psychopharmacology (Sousa et al., 2006). Several excellent computer solutions are available for specialized behavioral laboratories wishing to invest in infrastructure or in customizing open-source algorithms that are already available. Kinoscope, being an open source freely available program for behavioral pharmacologists, as well as other neuroscientists performing behavioral experiments, provides a basic but viable alternative. In our experience, the adoption of this software tool happens without imposing any burden on the day-to-day operations of a research team. Moreover, experienced staff members using the Kinoscope can streamline and audit the training of new members, by making use primarily of the visual maps, thus improving the consistency and reproducibility of scoring by novice researchers. Recently several concerns have been raised with regards to the validity of experimental data (Steckler, 2015; Bespalov et al., 2016). Many factors should be taken into account in improving the quality of experimental studies (Kilkenny et al., 2009; McNutt, 2014; Macleod et al., 2015) and perhaps another overlooked factor is the quality of manual scoring of behavioral experiments, which in turn may result in poor inter-rater agreement and inevitably low reproducibility. In our experience (Kokras et al., 2014, 2015, 2017), using Kinoscope's visual maps as visual aids, either in real-time scoring or in later offline auditing, greatly enhanced in an efficient and engaging way the training of new student members and the troubleshooting of poor reproducibility. Positive feedback has also been received from other departments that have used the beta version of this program, and several groups have already used the program for their research (Castelhano-Carlos et al., 2014; Papazoglou et al., 2015; Wiersielis et al., 2016; Lopes et al., 2017; Caetano et al., in press). The program will be under active development, with more behavioral templates scheduled for inclusion soon (Y-maze, Light/Dark, Tail Suspension Test). Additionally, as data transparency and data sharing has been proposed as a remedy for poor data reproducibility (Steckler et al., 2015), a possibility to export, import and exchange results and raw data produced by Kinoscope will be added. In the same context, the open-source code is also available for inspection and possible modifications (e.g., adding more behavioral templates by other research groups) at github.com. The authors also welcome any suggestions for future improvements. Availability of the latest version of the program is through the Sourceforge repository at https://sourceforge.net/projects/kinoscope, and a training video is also available at the same site along with a manual.

## Author contributions

NK and CD conceptualized, designed and led the development of the program. DB and FT wrote the software code. All authors contributed to the writing of the manuscript and approved the final version.

## Funding

This study has been funded by an IKY Fellowship of Excellence for Postgraduate Studies – Siemens Program to NK. The costs for this open-access publication are supported by the ECNP Network “Preclinical Data Forum” (https://www.ecnp.eu/projects-initiatives/ECNP-networks/List-ECNP-Networks/Preclinical-Data-Forum.aspx). The ECNP Network “Preclinical Data Forum” neither promotes nor endorses the use of the software tool reported in this publication.

### Conflict of interest statement

NK has received honoraria and travel support from Janssen-Cilag, Lundbeck, Sanofi-Aventis, Medochemie Generics and Elpen S.A. CD has received honoraria from Janssen-Cilag and travel support from Boehringer Ingelheim. The other authors declare that the research was conducted in the absence of any commercial or financial relationships that could be construed as a potential conflict of interest.

## Supplementary material

Supplemental Figure S1 **Screenshot of the computer dialog window for defining test templates**.

Supplemental Figure S2 **Screenshot of the computer dialog window for defining desired keystroke combinations**.

Supplemental Figure S3 **Screenshot of the computer dialog window for creating a new project**.

Supplemental Figure S4 **Screenshot of the computer dialog window for the input of experimental subjects**.

Supplemental Figure S5 **Screenshot of the computer dialog window for scoring behavior**.

Supplemental Figure S6 **Screenshot of the computer dialog window for exporting results**.
