# Supplementary material for: Kinoscope: An Open-Source Computer Program for Behavioral Pharmacologists
Source: Front Behav Neurosci. 2017 May 12;11:88. doi: 10.3389/fnbeh.2017.00088 (PMC5427106; doi:10.3389/fnbeh.2017.00088)
Supplement: Supplemental Figure S1 — Screenshot of the computer dialog window for defining test templates. [file Image1.PDF]

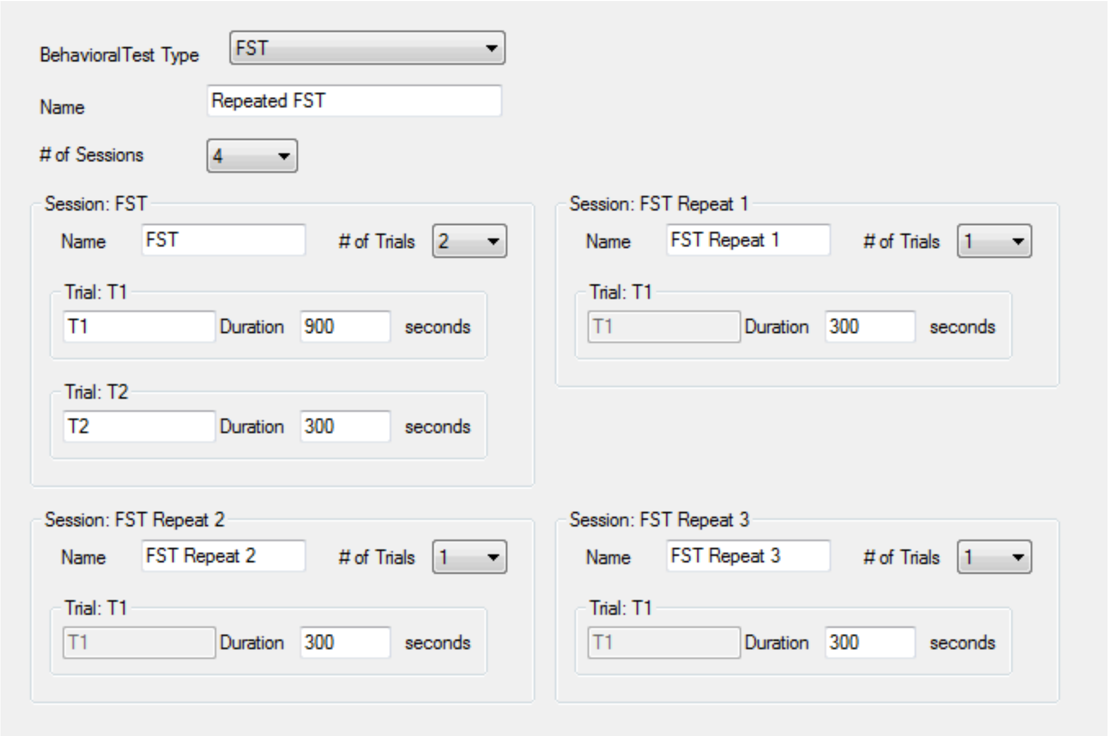

Suppl. Figure 1

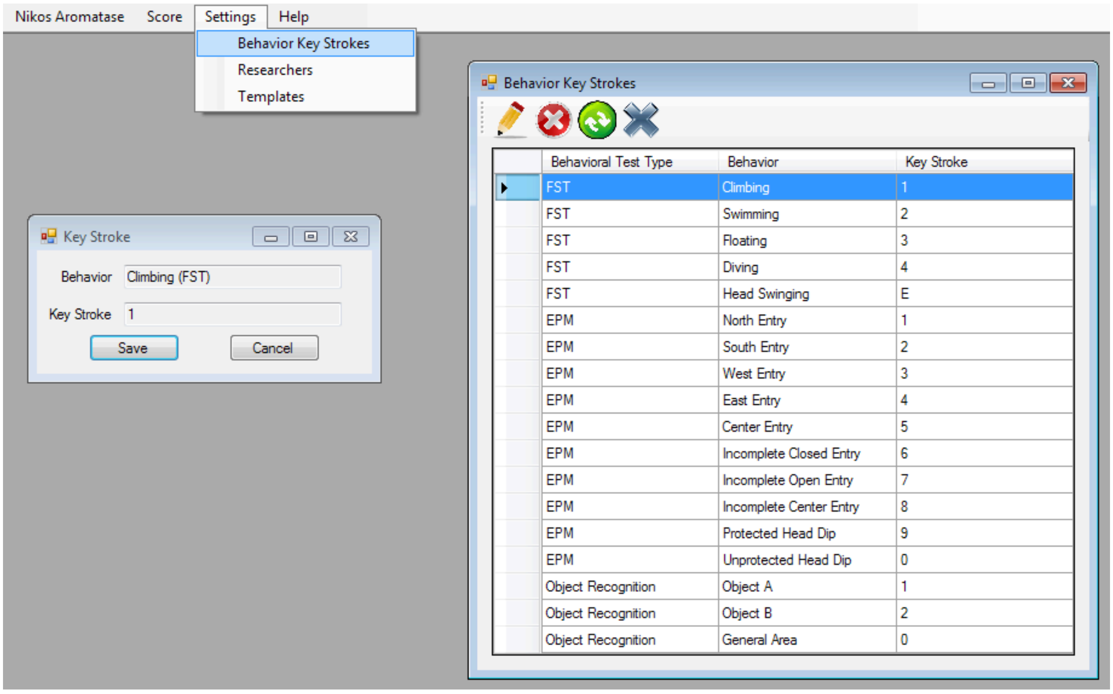

Supple Figure 2

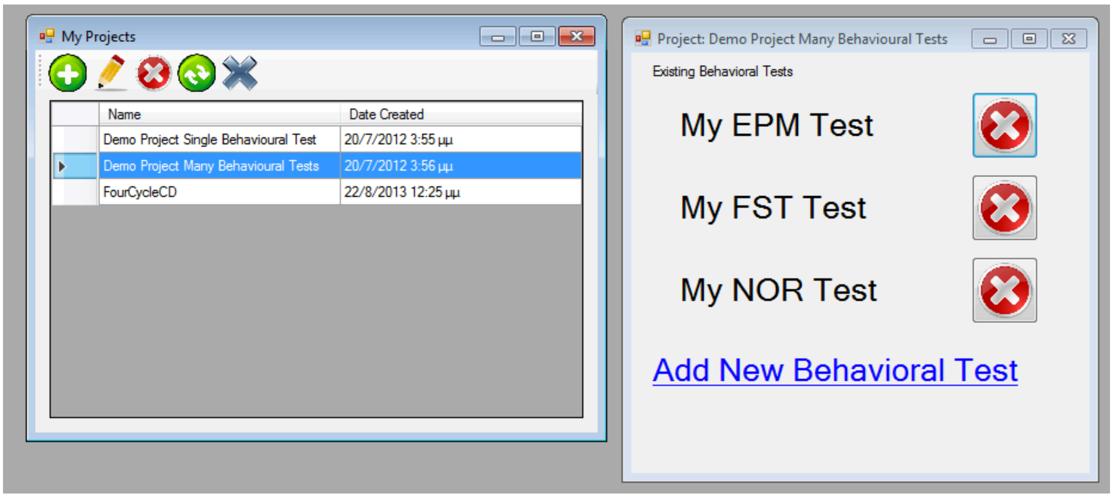

Supple Figure 3

Code	Subject Group	Strain	Sex	DOB
1	Control-Vehicle	Wistar	Male	8/9/2016 7:25 μμ
2	Control-Vehicle	Wistar	Male	8/9/2016 7:25 μμ
3	Control-Vehicle	Wistar	Male	8/9/2016 7:25 μμ
4	Control-Vehicle	Wistar	Male	8/9/2016 7:25 μμ
5	Control-Drug	Wistar	Male	8/9/2016 7:25 μμ
6	Control-Drug	Wistar	Male	8/9/2016 7:25 μμ
7	Control-Drug	Wistar	Male	8/9/2016 7:25 μμ
8	Control-Drug	Wistar	Male	8/9/2016 7:25 μμ
9	CMS-Vehicle	Wistar	Male	8/9/2016 7:25 μμ
10	CMS-Vehicle	Wistar	Male	8/9/2016 7:25 μμ
11	CMS-Vehicle	Wistar	Male	8/9/2016 7:25 μμ
12	CMS-Vehicle	Wistar	Male	8/9/2016 7:25 μμ
13	CMS-Drug	Wistar	Male	8/9/2016 7:25 μμ
14	CMS-Drug	Wistar	Male	8/9/2016 7:25 μμ
15	CMS-Drug	Wistar	Male	8/9/2016 7:25 μμ
16	CMS-Drug	Wistar	Male	8/9/2016 7:25 μμ

Supple Figure 4

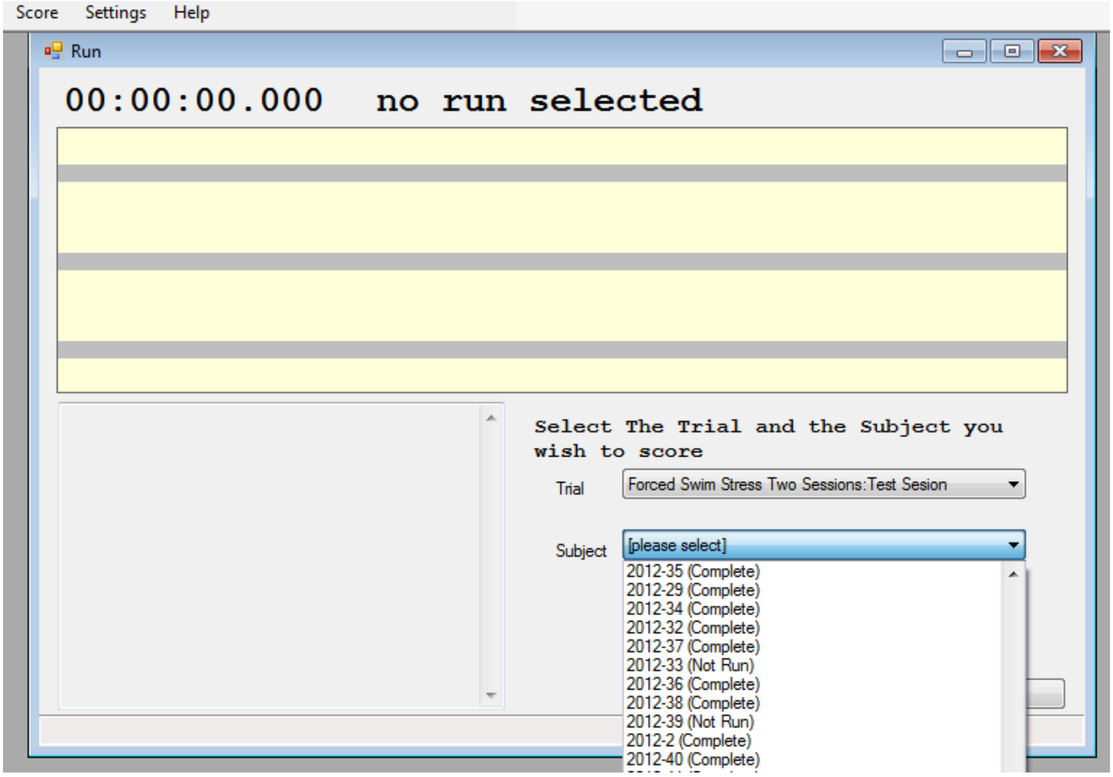

Suppl Figure 5

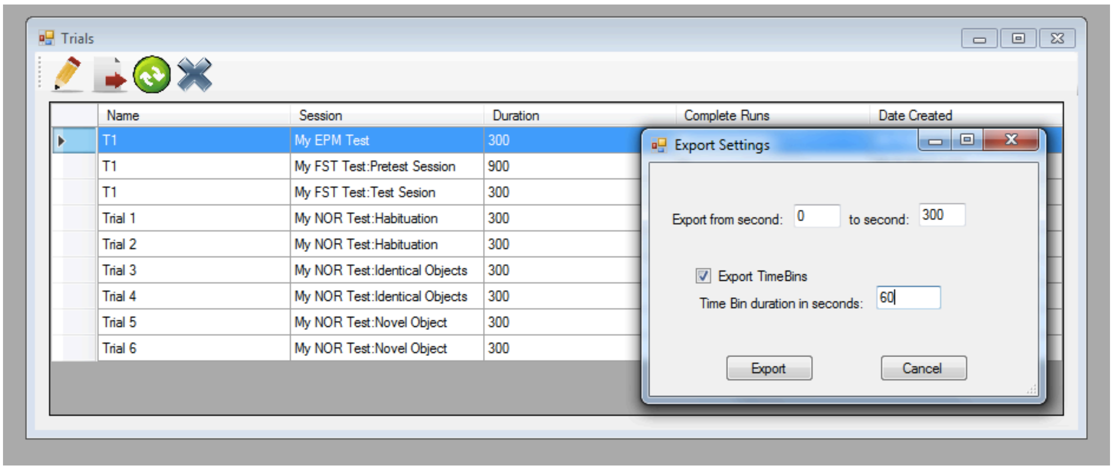

Trials
Name
Session
Duration
Complete Runs
Date Created
T1
My EPM Test
300
T1
My FST Test:Pretest Session
900
T1
My FST Test:Test Sesion
300
Trial 1
My NOR Test:Habituation
300
Trial 2
My NOR Test:Habituation
300
Trial 3
My NOR Test:Identical Objects
300
Trial 4
My NOR Test:Identical Objects
300
Trial 5
My NOR Test:Novel Object
300
Trial 6
My NOR Test:Novel Object
300
Export Settings
Export from second:
0
to second:
300
Export TimeBins
Time Bin duration in seconds:
60
Export
Cancel

Supple Fig 6
